# Integration of Digital Pathologic and Transcriptomic Analyses Connects Tumor-Infiltrating Lymphocyte Spatial Density With Clinical Response to BRAF Inhibitors

**DOI:** 10.3389/fonc.2020.00757

**Published:** 2020-05-14

**Authors:** Arturas Ziemys, Michelle Kim, Alexander M. Menzies, James S. Wilmott, Georgina V. Long, Richard A. Scolyer, Larry Kwong, Ashley Holder, Genevieve Boland

**Affiliations:** ^1^Department of Nanomedicine, Houston Methodist Research Institute, Houston, TX, United States; ^2^Department of Surgery, Massachusetts General Hospital, Boston, MA, United States; ^3^Department of Medical Oncology, Westmead Hospital, Westmead, NSW, Australia; ^4^Melanoma Institute of Australia, The University of Sydney, Sydney, NSW, Australia; ^5^Sydney Medical School, The University of Sydney, Sydney, NSW, Australia; ^6^Department of Tissue Pathology and Diagnostic Oncology, Royal Prince Alfred Hospital and New South Wales Health Pathology, Camperdown, NSW, Australia; ^7^University of Texas MD Anderson Cancer Center, Houston, TX, United States; ^8^Department of Surgery, Houston Methodist Hospital, Houston, TX, United States

**Keywords:** melanoma, targeted therapy (TT), immune infiltrate, spatial analysis, RNAseq

## Abstract

Metastatic melanoma is one of the most immunogenic malignancies due to its high rate of mutations and neoantigen formation. Response to BRAF inhibitors (BRAFi) may be determined by intratumoral immune activation within melanoma metastases. To evaluate whether CD8+ T cell infiltration and distribution within melanoma metastases can predict clinical response to BRAFi, we developed a methodology to integrate immunohistochemistry with automated image analysis of CD8+ T cell position. CD8+ distribution patterns were correlated with gene expression data to identify and quantify “hot” areas within a tumor. Furthermore, the relative activation of CD8+cells, based on transcriptomic analysis, and their relationship to other CD8+ T cells and non-CD8+ cells within the tumor suggested a less crowded distribution of cells around activated CD8+ T cells. Furthermore, the relative activation of these CD8+ T cells was associated with improved clinical outcomes and decreased tumor cell proliferation. This study demonstrates the potential of digital pathomics to incorporate immune cell spatial distribution within metastases and RNAseq analysis to predict clinical response to BRAF inhibition in metastatic melanoma.

## Introduction

The immune system is known to play a critical role in cancer pathogenesis, prognosis, and response to therapy (1–3). Indeed, activation of the immune system is critical for initiating tumor cell recognition and death, with or without therapy (4). However, immune response to cancer varies widely from patient to patient, and the extent to which this variation correlates with successful treatment remains a complex question that depends on the type of therapy given, the pre-therapy tumor microenvironment, and the neoantigens that are available for immune cell recognition (5). Due in part to a high mutation and neoantigen formation rate, metastatic melanoma is one of the most highly immunogenic cancers with the ability to induce adaptive immune responses (6), making it an ideal cancer type in which to address the role of immune cells in therapy responsiveness.

Although the high immunogenicity of melanoma has led to its widespread success in being treated with immune checkpoint inhibitors (7), the role of the immune system in targeted therapy is less well-defined. Despite data suggesting immunotherapy after BRAF inhibitor (BRAFi) therapy is associated with a lower response rate (8, 9), there are data suggesting that early treatment changes with BRAFi may actually improve antitumor immune responses (10, 11) and expression of tumor antigens (12) suggesting a cooperative interplay between initial BRAFi-induced changes and intratumoral immune activation (13, 14). Relevantly, high pre-treatment immune signatures were recently identified to correlate with tumor regression in melanoma patients undergoing BRAFi therapy (15), based on reverse phase protein array (RPPA) and RNA sequencing analyses, suggesting that immune signatures might serve as baseline biomarkers for response to BRAFi. Nevertheless, it remains unclear how this knowledge can be translated into the clinical setting. We propose that cytopathologic assessments are practical approaches, but the initial suggestive biomarker findings must first be validated (16).

Here, we have analyzed tumor-associated cytotoxic T cell localization and density in metastatic melanoma tumors prior to BRAFi therapy. Utilizing immunohistochemistry (IHC), we explored the idea that not only cell densities, but also the spatial positioning of CD8+ T cells and tumor cells within samples might correlate with clinical outcomes. We showcase the use of automated cell position detection software with machine learning algorithms to achieve deep statistical analyses of metastases prior to treatment with targeted therapy. Assessment of RNAseq data in conjunction with IHC nominated several genes with previously undescribed correlations between cellular distribution/density, CD8+ cell localization, and clinical outcomes. Furthermore, analysis of known CD8+ cell activation markers relative to known exhaustion markers revealed correlations with clinical outcomes that were independent of CD8+ cell density.

## Materials/Methods

### Patient Cohort/Samples

Fifty-two patients with metastatic melanoma treated at two institutions were included for analysis: Massachusetts General Hospital (MGH) and the Melanoma Institute Australia (MIA). Patients included were > 18 years, had a diagnosis of metastatic melanoma (unresectable stage III or stage IV), and were treated with targeted therapy at standardly approved doses. IRB approval was obtained at each institution prior to study enrollment and written informed consent was obtained from patients prior to analysis. Accessible tumors amenable for biopsy were sampled from metastatic sites prior to treatment with BRAFi or combination BRAFi plus MEK-inhibitor (MEKi). A separate informed consent document was signed for all additional procedures. Patient characteristics are in Supplemental Table 1.

### Immunohistochemistry

Slides of patients with a known BRAF V600E mutation were obtained from the Massachusetts General Hospital (*n* = 17) and the Melanoma Institute Australia (*n* = 35) and underwent staining for CD8+ T cells at pre-treatment time points. Slides were scanned using the NDP Nanozoomer HT (Hamamatsu Photonics). After formal dermatopathology review, the whole slide images were digitized using the Hamamatsu NDP viewer and tumor area was annotated. Whole slide images (.npdi) and corresponding tumor annotations (.xml) were imported to Definiens Tissue Studio (Definiens, Inc). Nuclei were segmented using hematoxylin stain and simulated membrane. We identified CD8+ cells, which were DAB or AP Red positive cells. For further spatial analysis, x- and y-coordinates of each individual cell were exported, cell spatial analysis was accomplished as described in the integrative data analysis section.

### Sequencing of mRNA

Transcriptome sequencing principles and its utility to assess gene expression have been previously described (17). Data was generated based on Illumina chemistry with mean sequencing quality = 37, library insert size = 150 bp, mapping rate = 99%, and expression profiling efficiency = 0.79 as previously described (15). RNA-seq data were previously deposited in the European Genome-phenome Archive (EGA S00001000992).

### Integrative Data Analysis

**Figure 1:**
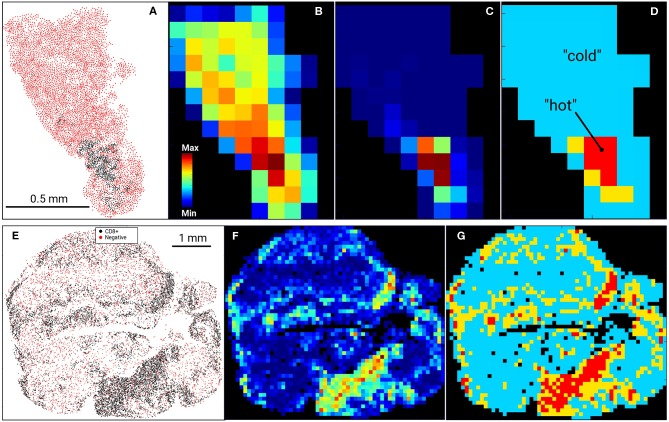
Digital Imaging Analysis. Examples of US (*top row*, **A–D**) and AU (*bottom row*, **E–G**) tissue samples and processing. Input data (**A**–US cohort; **E**–AU cohort) were tessellated into a rectangular 100 μm grid and cell heatmaps were calculated for all cells **(B)** or CD8+ cell density **(C,F)**. CD8+ heatmaps were used to segment tissue into CD8+-cold (low density) and CD8+-hot (high density) areas, where %HOT and %COLD denotes the area fractions of tissues corresponding to CD8+-hot and cold segments relative to the entire tissue heatmap **(D,G)**. **(D)** designates “hot” as red, “cold” as blue and thus %HOT = (area of “hot segments)/(area of all segments of tissue on grid).

**Figure 2:**
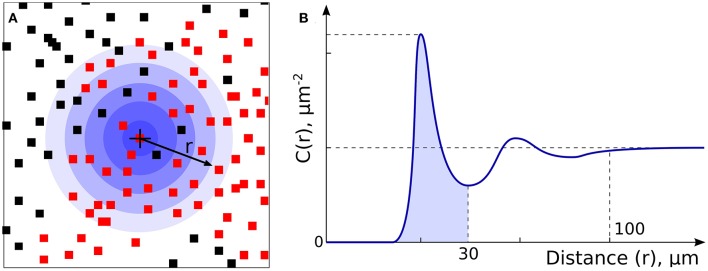
Non-CD8+ and T cell Spatial Relationships. **(A)** Non-CD8+ cells (NCs, black dots) and T cells (red dots) were analyzed to allow assessment of density and spatial relationships. **(B)** Analysis of radial cell concentration, C(r), allows assessment of the concentration of cells of certain type around CD8+ or NC.

Cartesian coordinates of CD8+ and other (cells other than CD8+; referred as non-CD8+ cells or NC) cells were used to calculate a CD8+ cell density heatmap with a 100 μm grid in a tissue tessellation process, as illustrated in Figures 1, 2. Non-tissue grid areas were excluded from analysis. By using CD8+ heatmaps, tissue areas with highest CD8+ cell density were designated as “hot” areas and the percentage of this “hot” area relative to the entire tissue area on the grid was designated as %HOT. Next, spatial and descriptive statistics of CD8+ cells were separately analyzed in the entire tissue grid and in %HOT area and then correlated with clinical outcomes and RNAseq data. Cell spatial distribution analysis was accomplished with custom scripts in Python (www.python.org) and libraries *numpy* (numpy.org; ver. 1.17) and *scipy* (www.scipy.org; ver. 1.4).

All gene expression data (~55,000 genes) of patients from RNAseq analysis was correlated to clinical outcomes and spatial CD8 properties with a Python script. All genes were used because the ratio of genes may define biological state of CD8 cell for which we have to rely on all available gene expression data. Each correlation instance was accomplished by using numpy Python library and its *numpy.corrcoef()* function, which calculated Pearson correlation coefficients. Because the function returns a correlation coefficient matrix, only the non-diagonal elements were used to characterize the correlation between a gene transcription and other clinical characteristics. Then, the calculated correlation tables were assembled in LibreOffice (www.libreoffice.org). “The statistical significance for plotted correlations was calculated based on student's *t*-test statistics using SigmaPlot software (systatsoftware.com/products/sigmaplot/).

## Results

### Digital Imaging Allows Single-Cell Resolution of Non-CD8+ Cells (NCs) and Tumor-Infiltrating CD8+ Cells

In our previous work (15), RNA levels of CD8A and CD8B (markers of cytolytic T cells) in tumors from patients prior to initiating BRAFi therapy demonstrated a positive correlation with eventual tumor regression after treatment. To validate this data at the protein level, we utilized immunohistochemistry to assess non-CD8+ cell (NC) and CD8+ cell densities in pre-treatment biopsies from two separate melanoma cohorts, one from the Massachusetts General Hospital (US) and one from Melanoma Institute Australia (AU). Slides were digitally scanned and CD8+-negative cells (NCs) and CD8+-positively-staining cells were identified and assigned positional coordinates, creating a tissue-specific heatmap amenable to deep statistical assessment. Figure 1 provides examples of US and AU slides and processing. Using the cell coordinates (Figures 1A,E), samples were tessellated in a rectangular grid, allowing us to calculate the means and standard deviations of cell densities. Furthermore, this allowed the creation of cell heatmaps (Figures 1B,C,F) and automatically segmented tissue into “CD8+-cold” and “CD8+-hot” areas (Figures 1D,G). Non-labeled cell (NC) and CD8+ T cells were enumerated and evaluated for cellular density relationships and spatial distribution across the tumors (Figure 2). The initial analysis focused on enumeration of cellular populations, dividing samples into non CD8+ cells (NCs) and CD8+ T cells for comparison across the two cohorts.

### CD8+ Spatial Distribution Is a Clinically Meaningful Parameter and Reflects Patient-Specific Tumoral Heterogeneity

**Figure 3:**
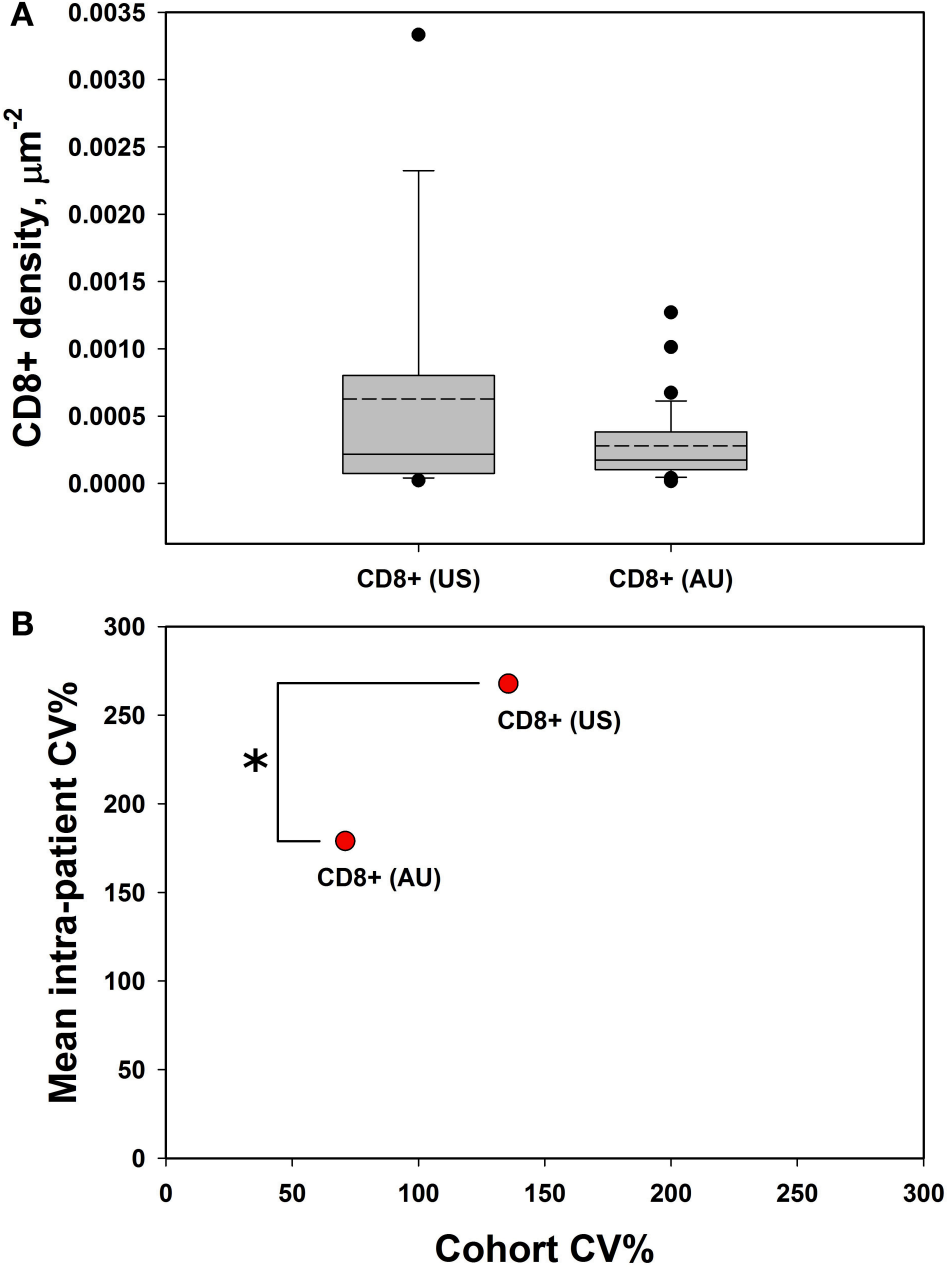
CD8+ cells demonstrate high intra-tumoral heterogeneity. **(A)** CD8+ cell densities are similar across the US and AU cohorts. **(B)** There was greater intra-patient heterogeneity than cohort heterogeneity across both cohorts (AU and US cohorts differ by intra-patient CV%; *p* = 0.014 by student's *t*-test), suggesting that CD8+ cell density may be a meaningful clinical variable for analysis. *p < 0.05, CV = coefficient of variance.

Our initial analysis of CD8+ cell counts did not show correlation with clinical outcome measures including RECIST response, progression-free survival (PFS), or overall survival (OS) in either cohort (Supplemental Figure 1). We therefore explored other potential factors that could explain the lack of concordance between CD8+ T cell enumeration via IHC and our RNAseq correlations of T cell markers with clinical outcomes (15). We hypothesized that the spatial distribution of CD8+ T cell might be a statistically useful parameter rather than considering only CD8+ cell counts. Specifically, we noted that some samples showed foci of high density, clustered CD8+ T cells vs. other samples that had more diffuse patterns of CD8+ T cells. Given that pockets of dense cytolytic activity may produce different outcomes compared to CD8+ T cells with a more sparse and uniform localization, we proceeded to systematically examine density and localization of T cells within the samples. When samples were examined for both intra-cohort and intra-patient CD8+ heterogeneity, we found that CD8+ cell densities were similar in the two cohorts (Figure 3A). Intra-patient heterogeneity in CD8+ densities were more pronounced than inter-group variability (Figure 3A). In other words, there was a wide diversity in high-density CD8+ clusters vs. diffuse CD8+ T cell distribution patterns within individual patient tissue samples as compared to within cohorts. However, there is a significant difference in the mean intra-patient variability between the US and Australian (AU) cohorts of patients (Figure 3B). This finding suggests that CD8+ cells are distributed inhomogeneously and that this inhomogeneity is variable from patient to patient, making it a clinically meaningful parameter for further correlation analyses (18). It is clear that there are differences in BRAF mutation rates that vary geographically [i.e., higher rates of V600K/R vs. V600E in Australia, likely reflecting differential UV exposure (18, 26)]. In our cohort, 30% of the Australian patients had a BRAF V600K mutation, as compared to 12.5% of the MGH cohort. It is apparent that this higher UV exposure in the Australian cohorts can impact tumor mutational burden, number of tumor infiltrating lymphoctyes (TIL), and subsequent response to therapy. Overall, the Australian cohort may demonstrate differences in TIL, which will impact CD8+ variability and variability as seen in Figure 3.

### A Scoring System for T Cell Density and Spatial Distribution Allows Global Assessment of “Hot” and “Cold” Areas of a Tumor

**Figure 4:**
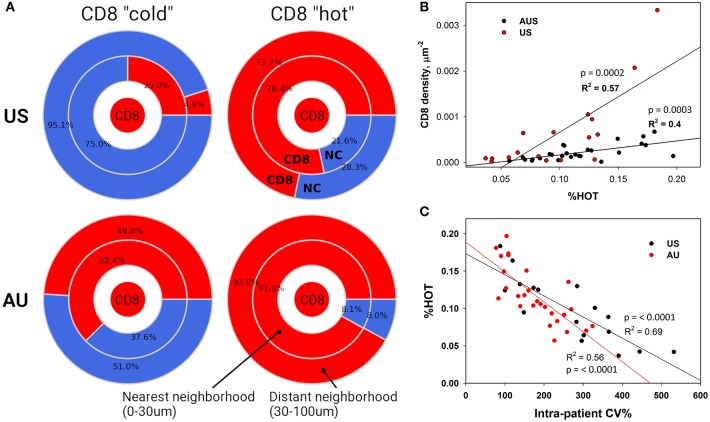
Neighborhood Analysis Defines CD8+ “Hot” and “Cold” Regions. **(A)** Cellular composition surrounding CD8+ cells was calculated for CD8+ hot and cold tissue segments. The numbers represent calculated percentages of cells (*red* – CD8+, *blue* – negative or “other” cells) composing the immediate (0–30 μm) and distant (30–100 μm) neighborhoods of CD8+ cells. **(B)** These densely packed CD8+ “hot” areas also correlate with higher numbers of infiltrating CD8+ cells and **(C)** lower CD8+ intra-patient heterogeneity.

The terms %COLD and %HOT were then used to denote the proportion of the tumor corresponding to the CD8+ depleted (“cold”) and CD8+-enriched “hot” areas. In summary, our approach allows us to investigate not only overall CD8+ infiltration, but also its heterogeneity and the pattern of local non CD8+ cell (NC) and CD8+ cell neighborhoods within individual tumors. Using our novel algorithm, each sample was segmented into CD8+-cold (low density) and CD8+-hot (high density) areas, and %HOT and %COLD were defined to denote the corresponding tissue area fractions (Figure 4). The segmentation algorithm enabled us to examine the CD8+ cell local microenvironment in greater depth (Figure 4A). The analysis starts with individual CD8+ cells and calculates cell-cell distances and radial cell concentration profiles. CD8+ cells are tightly surrounded by as much as 90% of other CD8+ cells (red) within CD8+-hot tissue areas and up to 95% non CD8+ cells (NC) in cold areas (blue). We find that %HOT is loosely correlated with CD8+ cell density (Figure 4B) and correlates with CD8+ T cell infiltration variance, supporting it as a readout of CD8+ heterogeneity (Figure 4C).

### Increased Relative T Cell Activation via Paired IHC and RNAseq Analysis Is Associated With Improved Clinical Outcomes

**Figure 5:**
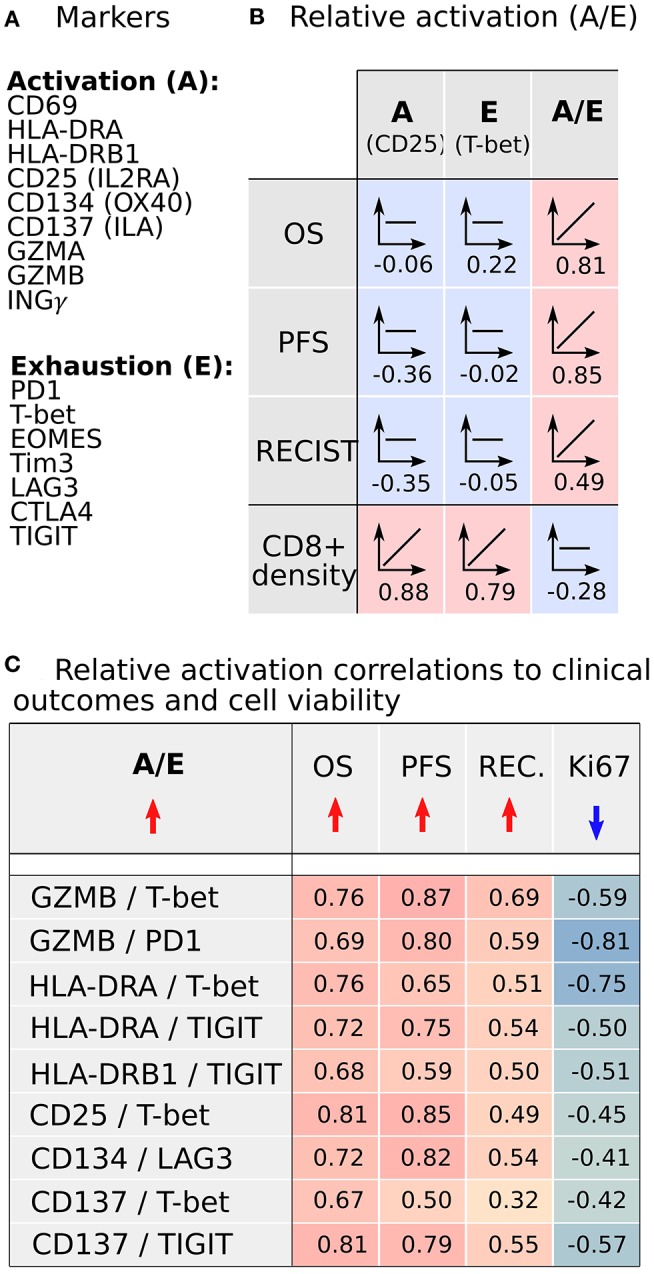
CD8+ activation and exhaustion transcriptome analysis from metastases demonstrates that increased relative CD8+ activation (A/E) correlates with improved clinical outcomes following BRAFi. **(A)** Transcriptomic signals of established CD8+ activation and exhaustion genes were analyzed. **(B)** Expression of individual activation or exhaustion genes correlated with CD8+ cell density but not clinical outcomes. However, the ratio of activation to exhaustion gene expression (A/E; CD8+ relative activation) directly correlated with clinical outcomes and was independent of CD8+ infiltration density. **(C)** Nine A/E ratios demonstrated better clinical outcomes (OS, PFS, RECIST) with increased A/E and reduced melanoma cell proliferation (Ki67).

We sought to understand if there are underlying properties of CD8+ infiltration in melanoma metastases that could affect clinical outcomes of BRAFi treatment; however, our initial analysis did not reveal any substantial support for this hypothesis. In fact, there was a lack of strong positive correlations between clinical outcomes and the absolute number of infiltrated CD8+ cells. However, following this unexpected result, we recognized our analysis may not characterize the functional status of CD8+ cells. Clearly, the immunological viability depends not only on the density of CD8+ cells but also on the biological activity of CD8+ cells, namely activation vs. exhaustion. Thus, we analyzed our RNAseq data for commonly accepted gene signatures of CD8+ activation (CD69, HLA-DRA, HL-DRB1, CD25/IL2RA, CD134/OX40, CD134/ILA) and exhaustion (PD1, T-bet, EOMES, Tim3, LAG3, CTLA4, TIGIT) (19). The activation gene panel was expanded to include granzymes (GZMA, GZMB) and interferon-gamma (INGγ), as the gene expression products responsible for the cytotoxic function of CD8+ cells (Figure 5A). The gene expression in both activated and exhaustion panels were positively correlated with CD8+ density in melanoma metastases, as illustrated by the activation marker CD25 and the Exhaustion marker T-bet (Figure 5B). These results are not unexpected and serve as an internal control, suggesting these gene signatures represent a true CD8+ cell population. However, gene expression for individual activation or exhaustion markers did not correlate with survival.

**Figure 6:**
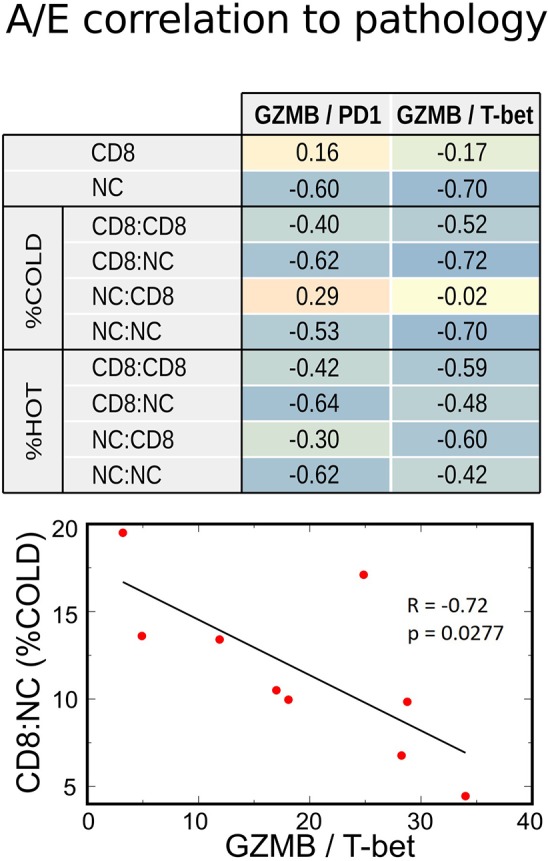
Correlations between transcriptome and IHC spatial immune profiling. Increasing relative CD8+ activation (represented by GZMB/PD1 or GZMB/T-bet ratios) strongly correlated with decreased Negative Cell (NC) density, especially in metastases with poor CD8+ infiltration (%COLD). This inverse correlation was also noted with NC clustering among themselves (NC:NC) and around CD8 + cells (CD8:NC). Therefore, a high CD8+ A/E ratio was associated with lower density of cell packing in tumors.

Since the functional status of CD8+ cells depends on the balance between activation and exhaustion, we analyzed the activation-exhaustion ratio (A/E), which we termed the Relative Activation, for the aforementioned A/E gene pairs. With this approach, we incorporated the effect of tumor-infiltrating immune cells in a more complete manner. Each relative activation was directly correlated to OS, suggesting that specific activation and exhaustion marker pairs are strongly related to clinical outcomes (Figure 5C). A/E correlation analysis indicated that the increasing A/E ratios (increasing activation, decreasing exhaustion, or both) was associated with reduced cell proliferation based on Ki67 staining (Figure 5C) and with increased OS (Figure 5C; range of *R* = 0.67–0.81), PFS (*R* = 0.50–0.87), and RECIST (*R* = 0.32–0.69) after BRAFi therapies in the US cohort. Interestingly, relative activation of GZMB showed strong inverse correlations to NC packing, including around CD8+ cells (Figure 6), suggesting that spatial distribution of activated CD8+ cells relative to other CD8+ cells and negative cells may affect lethality of cytotoxic T cells, specifically that suppressive effects of exhausted CD8+ cells may decrease the cytotoxicity of activated CD8+ T cells (CTL), despite these CTLs being in close proximity to negative cells that undoubtedly include tumor cells. While this data supports a direct correlation between clinical outcomes, transcriptome, and cellular spatial organization in tumors, there are limitations to the conclusions that can be made from our study. Notably, identifying whether cellular spatial organization or gene expression is the most significant factor in determining not only activity of CD8+ cells but also clinical outcomes necessitates a larger future investigation to assess causality.

## Discussion

In this study, we probed the question of whether unbiased analysis of non CD8+ cell (NC) and CD8+ cytopathologic characteristics can provide prognostic information relating to melanoma clinical responses to BRAF-inhibitor therapy, similar to approaches that have been used to assess the role of spatial distribution of CD8+ cells in the clinical outcome of patients with treatment-naive melanoma (20). In that work, spatial location of CD8+ cells but not relative percentage was directly correlated with overall and disease-free survival. The authors proposed that anti-cancer immune response requires direct interactions between CD8+ cells and malignant melanocytes, a justification for the meticulous image analysis to describe the distances between cells and provide a more complete representation of the intratumoral immune microenvironment. Besides melanoma, studies of other tumor types have suggested the importance of immune cell spatial distribution in clinical outcome. One study of pancreatic cancer tissue samples identified that increased T-cell infiltration (high vs. low), and also specifically cytotoxic T cell infiltration, was associated with increased overall survival (21). Furthermore, the proximity of cytotoxic T cells to cancer cells significantly correlated with increased overall survival. That study specifically noted that markers of T-cell activation could provide additional insight into the response to immunotherapy of pancreatic cancer compared to melanoma. By overlapping RNAseq data with IHC and computational image processing analysis, another study of both pancreatic cancer and head and neck squamous cell carcinoma primary tumor samples concluded that immune context could be used to predict therapeutic responses (22). Likewise, we identified that CD8+ distribution patterns (i.e., amount of “hot” tumor) correlate with gene expression data in the same cohort. These simple cytopathologic characteristics may serve as statistically robust parameters and create an early blueprint for how such parameters can be calculated and applied using machine-learning algorithms for detection, mapping, and quantification of immune cell infiltrates in melanoma metastases. In future studies, we envision applying this approach (i.e., %HOT) to larger panels of tumor and immune cell markers and with additional defining parameters such as location relative to the tumor-stromal borders.

Other studies have also demonstrated the importance of evaluating the phenotype of immune cells along with spatial distribution. In a study of primary melanoma tumors with spatial analysis of immune infiltration, another group similarly concluded that specifically activated cytotoxic T cells were prognostic, thus supporting our hypothesis that the activation status plays a critical role in clinical outcomes (23). In non-small cell lung cancer tumor samples, Barua et al. identified infiltration of Tregs, but not absolute CD8+ cells, to be a negative clinical marker yet increased infiltration of CD8+ cytotoxic T cells were capable of mitigating this effect (24). Similar to our findings that CD8+ density did not correlate with clinical outcomes and that relative activation may be a better prognostic marker, a study of pancreas tumors suggested that higher %Treg rather than absolute numbers of T cells was associated with decreased survival (25). Moreover, we similarly identified correlations between increased CD8+ activation/exhaustion ratio (Relative CD8+ activation) in melanoma metastases and improved survival and RECIST following BRAFi therapy, as well as decreased cell proliferation (Ki67 expression), suggesting that T cell activation status may not only predict clinical outcomes but also survival following BRAFi. Our results also underscore that the complexity of the tumor microenvironment necessitates combination variables including future studies with analysis of other immune cells within the tumor to develop a predictive spatial image biomarker.

Overall, these findings support the hypothesis that the immune system is a vital component in determining the effectiveness of targeted therapies on patient outcomes. Comprehensive studies are needed to dissect the immune contribution and identify potential synergistic immune-agonist approaches to combination therapy. In conclusion, through integration of two distinct data sets examining IHC staining of CD8+ cells in the setting of BRAF V600 mutant patients treated with MAPK-targeted therapy, we demonstrated that the absolute number of CD8+ T cells infiltrating into a melanoma metastasis does not strongly correlate with clinical outcomes, while density and spatial relationships offer biologically meaningful insights. Limitations of our study include gene expression data being available only for the US cohort and the inclusion of non-CD8+ immune cells in the “other” cells in our spatial analysis. However, our study is unique for its focus on metastatic melanoma tissue samples and on the comprehensive approach of spatial distribution and immune cell activation to predict response to BRAFi. The relative cell-cell distribution statistics can provide relationships to clinical outcomes and the cell transcriptome that were previously unappreciated, reinforcing the importance of multidisciplinary approaches to not only the rational design of patient therapies but also research discovery, even in the metastatic setting. Our study represents a step toward the incorporation of spatial coordinates to study the behavior and potential prognostic ability of tumor-infiltrating immune cells.

## Data Availability Statement

The datasets generated for this study can be found in the European Genome-Phenome Archive (EGA EGAS00001000992).

## Ethics Statement

The studies involving human participants were reviewed and approved by Dana Farber Harvard Cancer Center, Protocol 11-181 (PI: Boland). The patients/participants provided their written informed consent to participate in this study.

## Author Contributions

AZ led the imaging and RNAseq analysis, assisted in manuscript preparation and writing. MK was involved in data interpretation and manuscript writing. AM was responsible for material acquisition and data generation. JW, GL, and RS were material contributors and involved in manuscript preparation. LK was involved in hypothesis generation, data generation and interpretation, and manuscript preparation. AH was integral in data analysis and interpretation and manuscript preparation. GB was responsible for experimental planning, data generation, material contributions, data analysis, and manuscript preparation.

## Conflict of Interest

RS has received fees for professional services from Merck Sharp & Dohme, GlaxoSmithKline Australia, Bristol-Myers Squibb, Dermpedia, Novartis Pharmaceuticals Australia Pty Ltd, Myriad, NeraCare and Amgen. GB has sponsored research agreements with Takeda Oncology, Olink Proteomics, and Palleon Pharmaceuticals. GB has received fees for professional services from Novartis, NW Biotherapeutics, and Nektar Therapeutics. GL has served as an advisor or consultant to Aduro, Amgen, Bristol-Myers Squibb, Mass-Array, Merck, MSD, Novartis, OncoSec Medical, Pierre Fabre, Roche, and Sandoz. AM has served on advisory boards for BMS, MSD, Novartis, Roche, Pierre-Fabre. The remaining authors declare that the research was conducted in the absence of any commercial or financial relationships that could be construed as a potential conflict of interest.

## Abbreviations

RNA: ribonucleic acid
CD8: cluster of differentiation factor 8
RPPA: reverse phase protein array
RNAseq: ribonucleic acid sequencing
DAB - 3,3′: Diaminobenzidine
mRNA: messenger ribonucleic acid
NC: CD8+ negative cells
RECIST: Response evaluation criteria in solid tumors
PFS: progression free survival
OS: overall survival
A/E: activation to exhaustion ratio.
