# Supplementary material for: Integration of Digital Pathologic and Transcriptomic Analyses Connects Tumor-Infiltrating Lymphocyte Spatial Density With Clinical Response to BRAF Inhibitors
Source: Front Oncol. 2020 May 14;10:757. doi: 10.3389/fonc.2020.00757 (PMC7247820; doi:10.3389/fonc.2020.00757)
Supplement: Supplementary file 2 [file Data_Sheet_1.docx]

**Supplemental Figure**


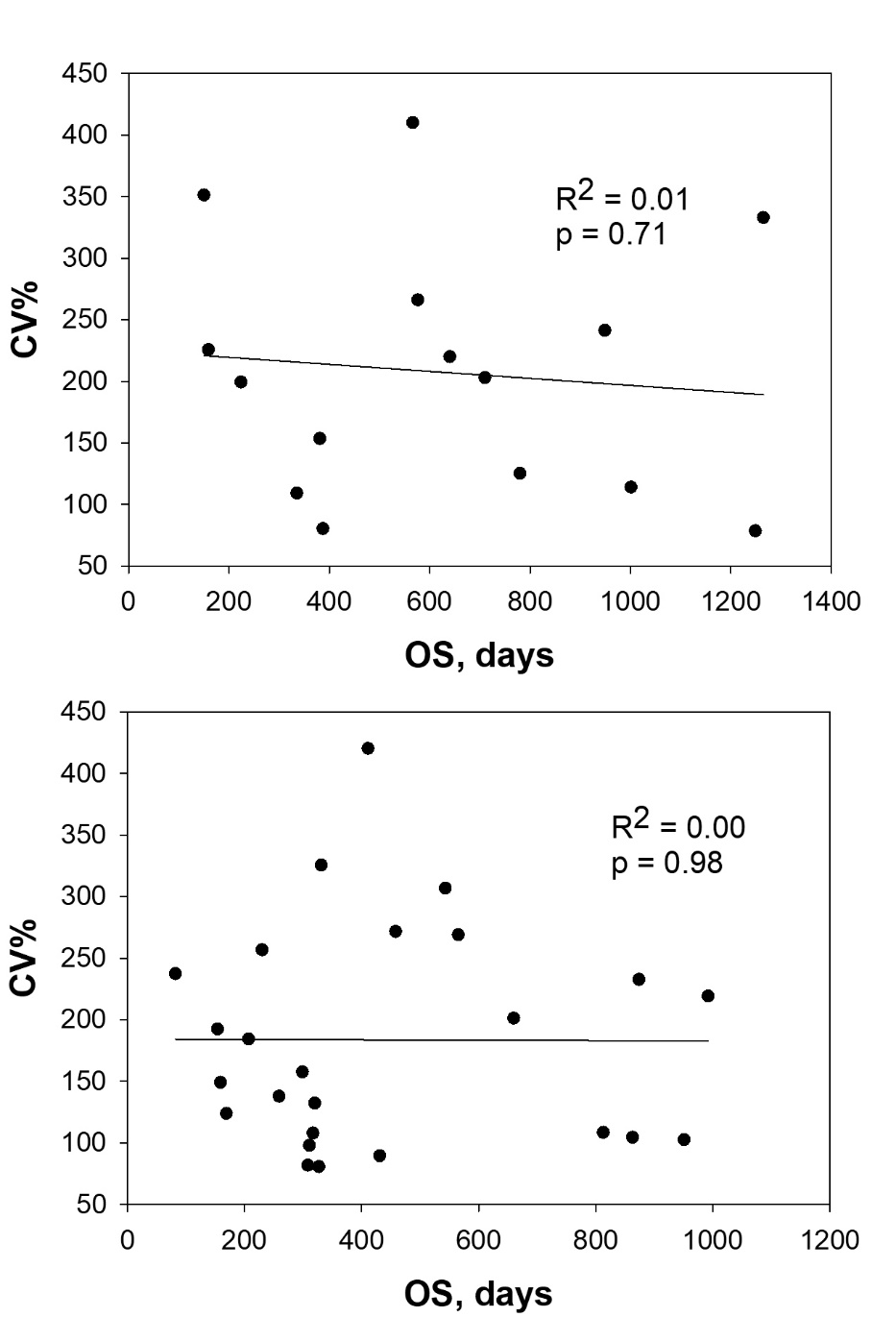


**Supplemental Figure 1.** Increased intra-patient variability in CD8+ density does not correlate with overall survival in the US (upper) nor the Australian (lower) cohort.
